# Pilot Study to Evaluate the Association Between the Length of the Luteal Phase and Estrous Activity Detected by Automated Activity Monitoring in Dairy Cows

**DOI:** 10.3389/fvets.2018.00210

**Published:** 2018-08-31

**Authors:** Jose Denis-Robichaud, Stephen J. LeBlanc, Andria Jones-Bitton, Bruna F. Silper, Ronaldo L. Aoki Cerri

**Affiliations:** ^1^Department of Population Medicine, University of Guelph, Guelph, ON, Canada; ^2^Applied Animal Biology, Faculty of Land and Food Systems, University of British Columbia, Vancouver, BC, Canada

**Keywords:** automated activity monitoring, dairy cows, estrogen receptor, estrus expression, progesterone

## Abstract

The ability of automated activity monitoring (AAM) systems to detect estrus is influenced by multiple variables. The luteal phase (LP) preceding estrus has been shown to be important for hormones release, and behavior during estrus in ruminants, but its impact on activity as measured by AAM systems has not been studied. The objective of this study was to investigate the impact of the length of the preceding LP on the intensity and duration of estrus as measured by AAM, and on the expression of estrogen receptor alpha (ERα) in the endometrium. A total of 60 cows between 46 and 53 days in milk were assigned to either a normal or a short length LP. Length of the LP was insured by the use of hormonal synchronization programs. Cows in both treatments were matched on parity, milk production, body condition score (BCS), and lameness score (assessed at enrolment). Expression of ERα receptors in the endometrium were evaluated by nuclear staining by immunohistochemistry of biopsies. Estrus was defined as the day on which the AAM system detected estrus. Cows that were not synchronized by the protocol or that were not detected in estrus by the AAM systems were excluded, which left 21 and 11 cows for analyses in the normal and short length LP, respectively. Peak activity index of estrus, duration of estrus, and expression of ERα were outcomes for multivariable linear regression models. Cows with short length LP tended to have lower peak activity at estrus, but there was no significant effect of treatment when BCS was accounted for. Cows with BCS ≤ 2.5 had less intense and shorter estrus than cows with BCS ≥ 2.75. There was no association between the length of the LP and the expression of ERα in the endometrium.

## Introduction

Automated activity monitoring (AAM) systems have been developed and refined as an estrus detection aid in dairy cattle (1–3). They are used on many dairy farms to manage reproduction and decrease the time spent on estrus detection (4, 5). The ability of a system to identify cows in estrus, and the probability of pregnancy for inseminations following an AAM system alert, have been shown to affect the system's economic value in reproduction management programs (6, 7). Previous studies found the sensitivity of a collar-mounted AAM systems (Heatime®, H-Tags, SCR Engineers, Netanya, Israel) varied between 52 and 72%, and its positive predictive value between 64 and 94% (8–11), which is comparable to other systems (11–13). This variability of one system depends on what gold standard was used, but also on the threshold used, and the conditions it is used in (8–13). The ability and accuracy of an AAM system to identify cows in estrus do not depend only on the system itself. Cow factors such as parity, milk production, and body condition score (BCS), as well as concentrations of estradiol and progesterone (P4) at estrus have been shown to be associated with intensity and duration of estrus as detected by AAM systems, and can therefore affect the ability of the system to identify estrus (14–16). Multiparous cows have been shown to have less intense and shorter estrus than primiparous cows, as have thin cows (BCS ≤ 2.5) compared to normal and fat cows (BCS ≥ 2.75), and high-producing cows (≥39 kg milk/days) compared to low-producing cows [ ≤ 31 kg milk/days; (16)]. Parity and BCS are also reported to be associated with estradiol concentration at estrus, which can explain partially these associations (16). High estradiol and low P4 at estrus have been reported to be associated, although weakly, with intensity and duration of estrus as detected by AAM systems (15, 16).

Circulating estradiol concentration at estrus plays a key role in estrous behavior because it activates estrogen receptors in the area of the hypothalamus regulating sexual behavior (17–19). Low concentrations of P4 (because P4 blocks the effect of estradiol on the hypothalamus), and expression of estrogen receptors (ER) in the hypothalamus are necessary for estrus expression (19–21). The expression of ER in the hypothalamus has been shown to vary according to the stage of the estrous cycle and circulating concentrations of estradiol and P4 (18, 19, 22, 23). Moreover, exposure to P4 prior to estrus has been shown to be positively associated with intensity of estrus expression in ewes (24). The expression of ER in the reproductive tract of different species is reported to be regulated by circulating P4 (25, 26). Specifically, ER alpha (ERα) decreased during the luteal phase, likely due to P4 exposure, and reached its inflection point around days 14 of the cycle.

Implementing a controlled trial with binary reproductive outcome variables such as detection of estrus or not requires enrolling a high number of cows (27). Preliminary data is important to evaluate the feasibility of the trial by exploring a similar hypothesis and identifying potential problems and limitations. We hypothesized that the duration of exposure to P4 before estrus (i.e., the length of the LP) is associated with the intensity and duration of estrus, as detected by AAM systems, irrespective of the estradiol concentration at estrus. The objective of this pilot study was to evaluate the association between the length of the LP and the intensity and duration of estrus, as detected by AAM. A secondary objective was to compare the expression of ERα in the endometrium following LP of different length.

## Materials and methods

### Animals

**Figure 1:**
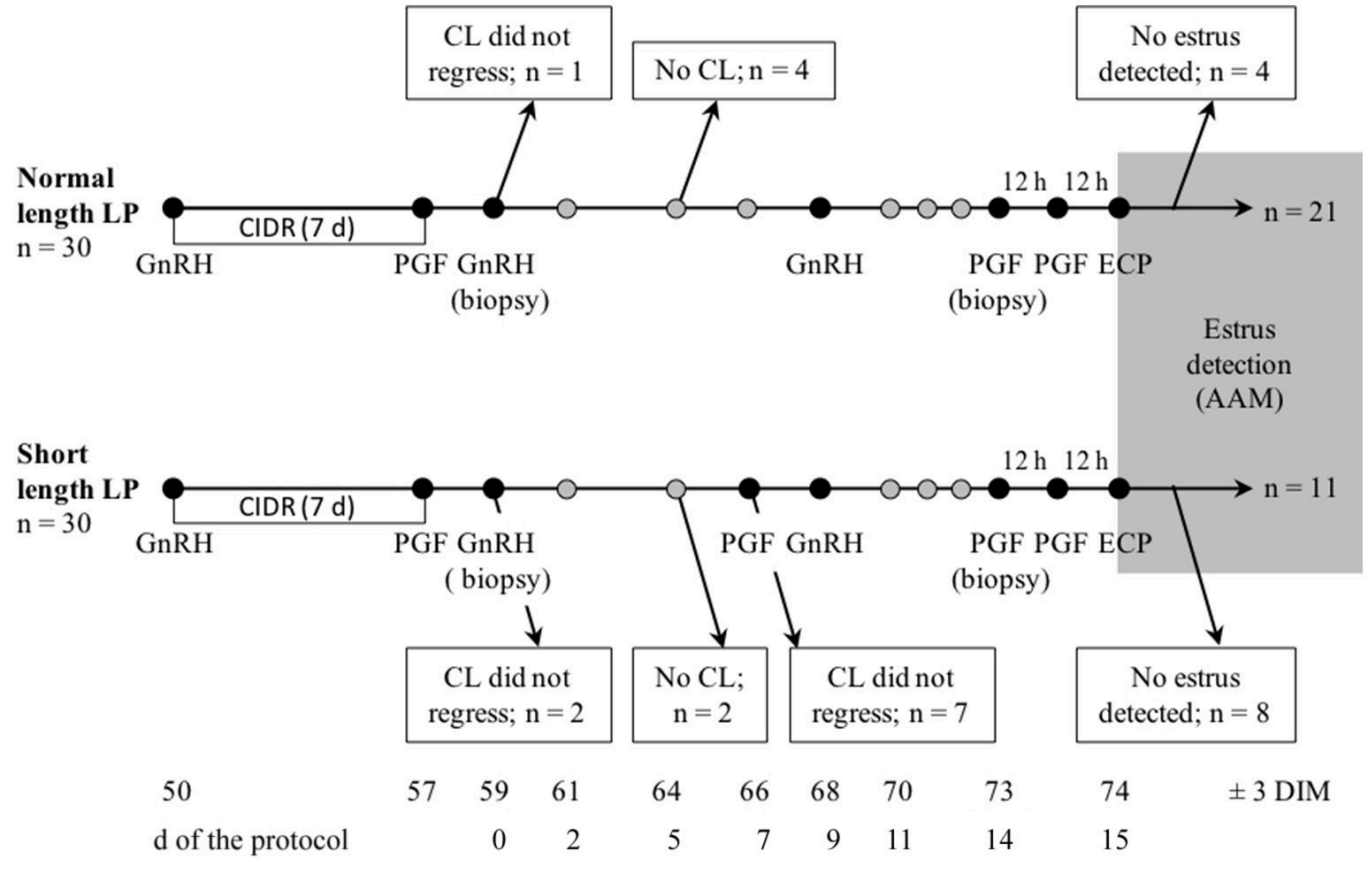
Schematic representation of sampling and treatments, and flow of the number of lactating cows enrolled in a controlled trial to compare estrus detected by AAM systems after a short or normal length luteal phase (LP). A total of 60 cows (30 per treatment) were enrolled at 46–53 DIM in one of two treatments: normal or short length LP. For all cows, a P4 vaginal implant (CIDR) was inserted and 100 μg of gonadorelin (GnRH) was administered. The implant was removed 7 days later and 500 μg of cloprostenol (PGF_2α_) was administered. Day 0 of the protocol was two d later when the second GnRH was administered. On 7 days of the protocol, cows with short length LP received PGF_2α_. All cows received GnRH on 9 days of the protocol, then two injections of PGF_2α_ 12 h apart on 14 days, and finally 0.5 mg of estradiol cypionate (ECP) on 15 days (12 h after the last PGF_2α_). Estrus expression after 14 days (gray area) was recorded using a collar-mounted activity system (Heatime®, H-Tags, SCR Engineers, Netanya, Israel). Samples for serum progesterone were taken on 0, 2, 5, 7, 9, days and daily from days 11 to estrus, as detected by AAM, and ultrasound of the ovaries was done on 2, 5, 9, 12, 14, days and daily from days 16 to estrus. On 0 and 14 days, endometrial biopsies were taken on a subsample of cows (*n* = 18).

This study was evaluated and approved by the University of British Columbia (UBC) Animal Care Committee (Protocol # 14-0019). Cows were enrolled in this controlled trial between 46 and 53 days in milk (DIM), were housed at the UBC Dairy Education and Research Centre (Agassiz, BC, Canada) in a free-stall barn, and stayed in the same pen throughout the study. At enrolment, cows were assessed for lameness using a standardized 5-point score [1 and 2: not lame, 3 and greater: lame; (28)] and for BCS using a 1 (emaciated) to 5 (fat) scoring system; (29). Parity and predicted 305 days milk production at first dairy herd improvement association (DHIA) test were obtained for all enrolled cows from DairyComp 305 (Valley Agricultural Software, Tulare, CA, United States). Cows were assigned to either the normal or short length LP (LP length was controlled by hormonal injections; Figure 1) such that they were matched in the two treatment groups by parity (first, second, or third and greater lactation), milk production quartile, BCS (≤ 2.5, 2.75–3.25, or ≥ 3.5), and lameness status (lame, or not lame).

### Treatments

To insure the protocol (summarized in Figure 1) started at 59 ± 3 DIM but did not begin with the first cycle of the cow, all cows received a vaginal implant containing 1.38 g of P4 (P4; CIDR, Zoetis, Kirkland, QC, Canada) and an intramuscular (i.m.) injection of 100 μg of gonadorelin (GnRH; Factrel, Zoetis, Kirkland, QC, Canada; 2 ml) at enrolment. After 7 days, the implant was removed, and cows received an i.m. injection of 500 μg of cloprostenol (PGF_2α_; Estrumate, Merck Animal Health, Kirkland, QC, Canada; 2 ml). The protocol started (0 days) 2 days later, when all cows received a second injection of GnRH (as above). Cows with a short length LP received an injection of PGF_2α_ (as above) on 7 days. All cows then received an injection of GnRH on 9 days, two injections of PGF_2α_ 12 h apart on 14 days, and finally 0.5 mg of estradiol cypionate i.m. (ECP; Estrus, Rafter8, Calgary, AB, Canada; 0.5 ml) on 15 days, 12 h after the last PGF_2α_. With these protocols, the LP preceding the estrus in the short and normal length LP were 5 and 14 days, respectively.

### Examinations, blood sampling, and analyses

Blood samples (10 mL) were taken on 0, 2, 5, 7, 9 days and daily from 11 days to estrus, from the coccygeal vessels into a sterile tube without anticoagulant (Vacutainer, Becton Dickinson, Franklin Lakes, NJ, United States). On 15 days, a blood sample (10 mL) was taken from the coccygeal vessels into a sterile tube with EDTA (Vacutainer, Becton Dickinson, Franklin Lakes, NJ, United States). Samples were kept chilled and allowed to clot. Within 5 h of blood collection, samples were centrifuged to harvest serum or plasma, which was frozen at −80°C. Serum P4 concentrations from 0, 2, 5, 7, 9, and 11 days to estrus were measured using a commercial ELISA kit Ovucheck Plasma; Biovet, St-Hyacinthe, Quebec; (30). This monoclonal antibody kit uses optical density of standards and serum samples read at 405 nm in a microplate absorbance reader. The range of quantification of the test is 0.55–10.45 ng/mL, and the intra-assay coefficient of variation (CV) in the present study was 11.7%. Plasma estradiol concentrations from 15 days were measured using radioimmunoassay [Perkin Elmer NET517250UC; Prairie Diagnostic Services, Saskatoon, SK, Canada; (31)]. This radioimmunoassay kit uses scintillation of charcoal-stripped serum standards and plasma samples after competitive binding with an immunoserum. The range of quantification of the test is 0–200 pg/mL, and the intra-assay CV in the present study was 17.0%.

Ovaries of all cows were scanned with a portable ultrasound (Ibex Pro; E.I. Medical Imaging, Loveland, CO) using a 7.5 MHz linear-array rectal transducer, on 2, 5, 9, 12, 14, days and daily from 16 days to estrus. Follicles and corpus lutea (CL) were examined from several angles and the largest cross-section of ovarian structures ≥5 mm in diameter were recorded. Luteolysis was defined as a *P4* ≥ 2 ng/mL on the day of a PGF_2α_ injection, followed by P4 < 2 ng/mL in the next 2 days. The threshold of 2 ng/mL was used as per the instructions in the ELISA manufacturer's insert (Biovet, St-Hyacinthe, Quebec), and because the serum samples were obtained early in the estrous period.

### Endometrial biopsies and immunohistochemistry

On 0 and 14 days of the protocol, endometrial biopsies were performed on a subset of cows (*n* = 9 per treatment). Epidural anesthesia was administered using 100 mg of lidocaine (Lidocaine HCl 2%, Vetoquinol, Lavaltrie, QC, Canada). The vulva was cleaned, and a disinfected guarded biopsy instrument (crocodile-type biopsy forceps, made by Aries Surgical, Davis, CA, United States) was introduced via the cervix into the body of the uterus by trans-rectal manipulation. A section of approximately 6 by 4 mm of the endometrium was taken on each d, then put for 24 h in 10% formalin solution (neutral buffered, HT501128, Sigma, Oakville, ON, Canada) at 4°C. Samples were then transferred to a 70% ethanol solution for storage at 4°C until they were dehydrated, embedded in paraffin wax, and sectioned for immunohistochemistry (IHC; Wax-It Histology Services, Vancouver, BC, Canada). Formalin-fixed paraffin-embedded sections of bovine endometrium were de-paraffinised and rehydrated with distilled water. They were retrieved in sodium citrate buffer (pH 6.0) via steamer and blocked with hydrogen peroxide in methanol and protein block to reduce background staining. Sections were then incubated with the primary antibody ERα (sc-7207; Santa Cruz Biotechnology, Dallas, TX, United States) at 1:100 or Rabbit IgG negative isotype control at 4°C overnight. The following day, the sections were incubated with the secondary antibody HRP Labeled Polymer Anti-Rabbit, developed with 3,3′-diaminobenzidine. They were then counterstained, dehydrated, and mounted.

**Figure 2:**
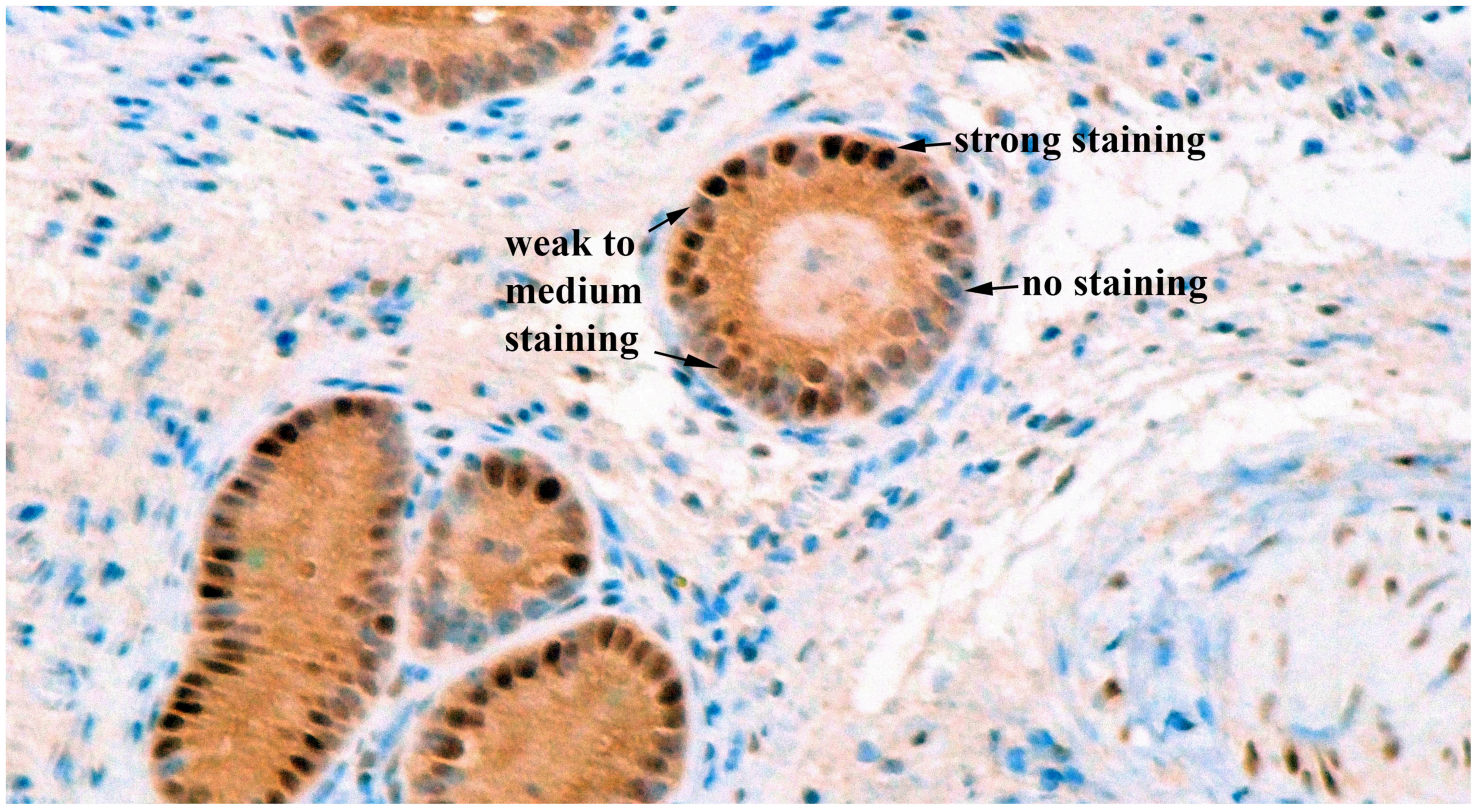
Immunohistochemical staining for estrogen receptor alpha in bovine endometrial glands at a 400X magnification. Blue cells were categorized as no staining, bluish-brown, and brown cells were categorized as weak to medium staining, and very dark brown cells were categorized as strong staining.

Image acquisition from the biopsy slides was done using Nikon Eclipse e200 microscope. If possible, 3 glands from different areas of the slide were selected. As shown on Figure 2, blue cells were categorized as no staining, bluish-brown, and brown cells were categorized as weak to medium staining, and very dark brown cells were categorized as strong staining (32). Counts of cells in each category were done at 400X magnification for each gland, and the proportion of cells in each category was calculated.

### Automated activity monitoring

Cows were fitted with a collar-mounted AAM system (Heatime®, H-Tags, SCR Engineers, Netanya, Israel), which continuously monitored individual cow activity using an accelerometer detecting movement and recorded average activity for 2 h periods. Data were transmitted every 2 h by a wireless system to the farm computer. The raw activity data were processed into an index value accounting for the baseline values of the previous 14 days. The index represents a value close to the SD of the raw data and ranges from 0 to 100 (larger numbers denoting higher activity). An estrous event was defined as a period when the index was above a threshold of 35 according to the manufacturer recommendation. The duration of estrus was defined as the time (sum of the number of 2 h periods) the activity index was above the threshold, and the peak activity index of estrus was the highest value during this period. Individual cow data (cow identification, date and time of the onset of estrus, peak activity of estrus, and duration of estrus) were extracted from the backup files using a macro in Excel (Microsoft Corporation, Richmond, WA, United States). The day of estrus was defined as the day on which the AAM system signaled an estrus.

### Statistical analysis

The sample size (*n* = 30 per treatment) was calculated to identify a difference of 10 points (*SD* = 14) in peak activity index of estrus with 95% confidence and 80% power, accounting for 30% loss to follow up (33). This sample size was also sufficient to identify a difference of 2.5 h (*SD* = 3.5) in duration of estrus. Cows that did not synchronize through the protocol (CL did not regress following the first PGF_2α_ or the second PGF_2α_ with short length LP, or no CL developed after the removal of the CIDR and the GnRH), and that did not have estrus detected by the AAM system were excluded from the analysis (Figure 1).

All statistical analyses were performed using SAS 9.3 (SAS Institute, Cary, United States). Descriptive statistics (PROC FREQ) for treatment, parity, milk production, BCS, and lameness score were calculated for all enrolled cows, excluded cows, and included cows with normal and short length LP. Peak activity index of estrus and duration of estrus were outcomes for linear regression models (PROC GLM). The proportion of cells in the 3 staining categories for glands were outcomes for linear regression models with repeated measures structure to account for the day of the biopsy (PROC GLIMMIX). In all models, treatment was included as a fixed effect. Even though matching was used to allocate cows to their treatment, the unequal pattern of excluded animals between treatment led us to include parity, milk production, BCS, and lameness score as confounders if the estimate for treatment changed by more than 10% when the covariate was removed (34, 35). Interaction terms between treatment and the other variables in the final model were retained if their *P* < 0.05. Assumptions of the models (normality and homoscedasticity) were assessed graphically using standardized residuals. Outliers (residuals and Studentized residuals), extreme (leverage), and influential data (Cook's distance and DFFITS) were used to assess the fit of the models (33). Marginal means (least squares means (LSM) ± SE) were obtained for categorical variables.

## Results

A total of 60 cows were enrolled in the study. Figure 1 represents the flow of animals throughout the study. Nine and 19 cows in the normal and short length LP, respectively, were excluded during the protocol (Figure 1). A total of 32 (53%) cows were identified in estrus and remained for statistical analyses. Table 1 presents the distribution of parity, milk production, BCS, and lameness score of cows enrolled, excluded, and included in the normal and short length LP.

**Table 1:** Characteristics of cows enrolled in a controlled study of the effects of a normal or short luteal phase (LP) on estrus expression.

		**Excluded**^**a**^		**Included**	
	**All enrolled cows n (%)**	**Did not synchronize n (%)**	**No estrus n (%)**	**Normal length LP n (%)**	**Short length LP n (%)**
n	60	16	12	21	11
**Parity**					
First	24 (40)	5 (31)	8 (66)	9 (43)	2 (18)
Second	11 (18)	2 (13)	2 (17)	4 (19)	3 (27)
Third and greater	25 (42)	9 (56)	2 (17)	8 (38)	6 (55)
**Milk production (305 days**^b^**)**					
< 11,063 kg	15 (25)	2 (13)	2 (17)	6 (29)	5 (46)
11,063–12,520 kg	15 (25)	3 (19)	3 (25)	6 (29)	3 (27)
12,520–13,593 kg	15 (25)	6 (38)	3 (25)	4 (19)	2 (18)
> 13,593 kg	15 (25)	5 (31)	4 (33)	5 (23)	1 (9)
**Body condition score**					
≤ 2.5	7 (12)	0	1 (8)	2 (10)	4 (36)
2.75–3.25	53 (88)	16 (100)	11 (92)	19 (90)	7 (67)
**Lameness score**					
Not lame (1–2)	49 (82)	12 (75)	12 (100)	16 (76)	9 (82)
Lame (3–5)	11 (18)	4 (25)	0	5 (24)	2 (18)

a *Cows were excluded from the analysis if their estrous cycle was not synchronized by the experimental protocol, or if they were not detected in estrus by the automated activity monitoring system*.

b *Predicted milk production for 305 d at first DHIA test*.

**Figure 3:**
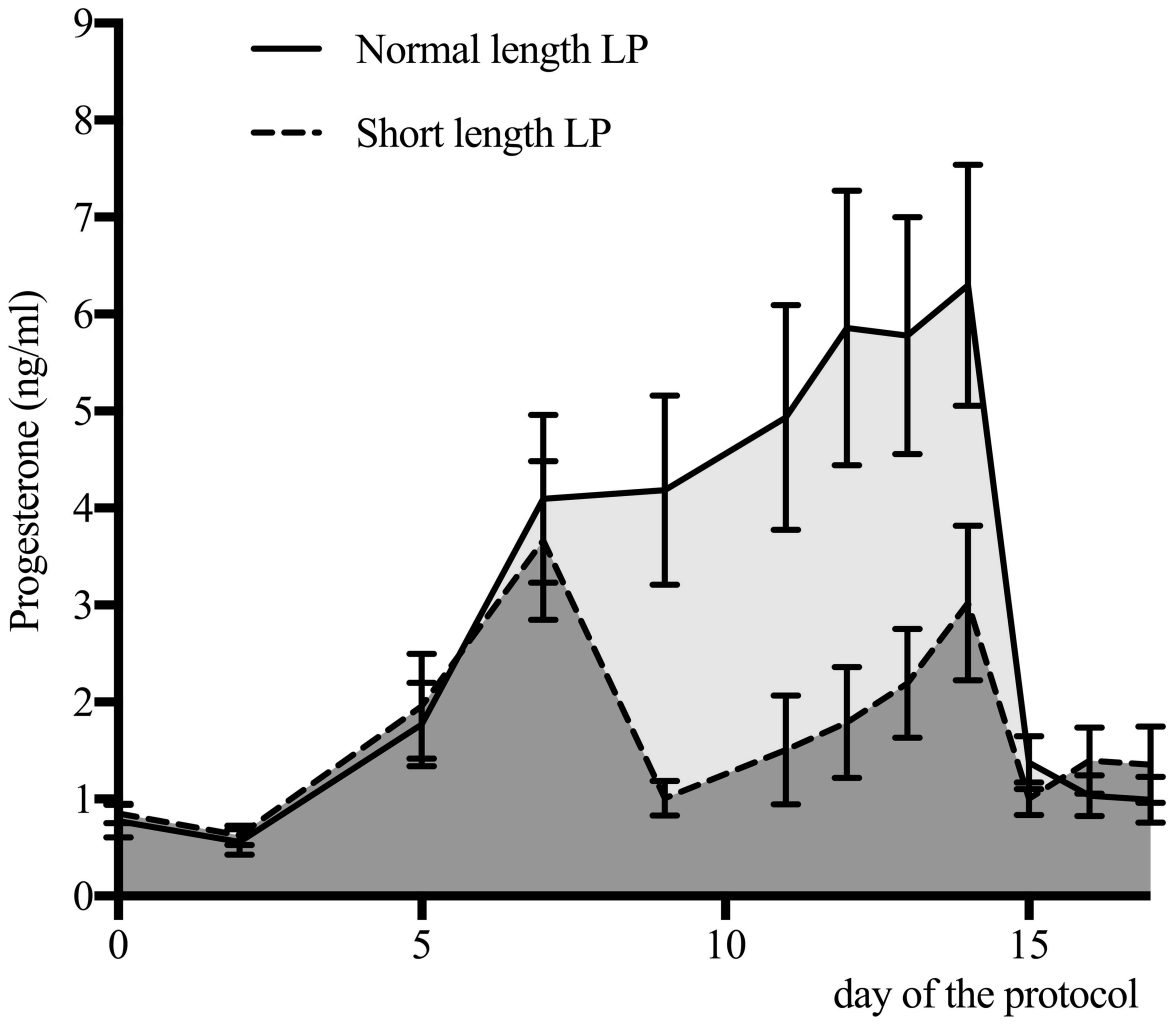
Serum progesterone concentration (mean ± 95% confidence intervals) for cows with a normal (*n* = 25) or a short length luteal phase (LP; *n* = 19). The area under the curve was 51.4 for the normal length LP (light gray area), and 29.0 for the short length LP (dark gray area).

**Figure 4:**
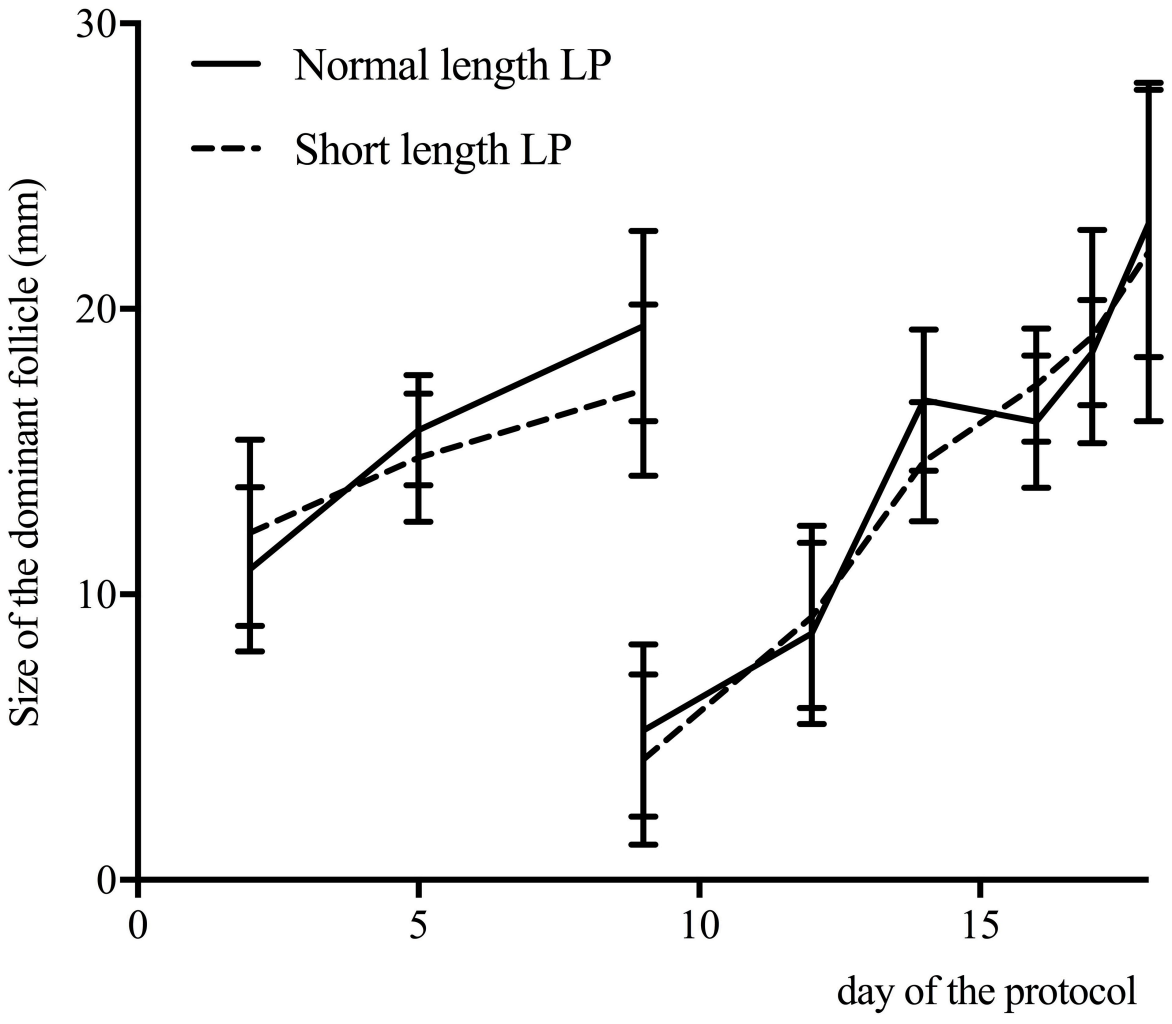
Size of the dominant follicle (mean ± 95% confidence intervals) determined by ultrasound of the ovaries on 2, 5, 9, 12, 14, days and daily from days 16 to estrus, for cows with a normal (*n* = 25) or a short length luteal phase (LP; *n* = 19). Cows ovulated before 16 days (normal length *LP* = 5; short length *LP* = 4), from 16 to 17 days (normal length *LP* = 4; short length *LP* = 7), from 17 to 18 days (normal length LP = 11; short length *LP* = 4), or after 18 days (normal length *LP* = 5; short length *LP* = 4).

Progesterone profiles for the normal and short length LP are presented in Figure 3. By design, the area under the curve was different between treatments (normal length LP: 51.4 ± 2.8; short length LP: 29.0 ± 3.6; *P* < 0.01). Progesterone at 9 days was 4.7 ± 1.8 ng/mL and 0.9 ± 0.4, in the normal and short length LP, respectively (*P* < 0.01). Progesterone at estrus (as detected by the AAM system) was 0.7 ± 0.1, and 0.9 ± 0.1 ng/mL in the normal and short length LP, respectively (*P* = 0.38). The follicular waves for the normal and short length LP are summarized in Figure 4. The size of the dominant follicles did not differ between treatments (*P* = 0.33). Estradiol at 15 days (before ECP; normal length LP: 3.3 ± 0.4 pg/mL; short length LP: 3.4 ± 0.4 pg/mL; *P* = 0.88), and dominant follicle diameter at 16 days (normal length LP: 19.5 ± 0.9 mm; short length LP: 18.3 ± 1.1 mm; *P* = 0.37) did not differ between treatments. Cows ovulated before 16 days (normal length LP = 5; short length LP = 4), from 16 to 17 days (normal length *LP* = 4; short length *LP* = 7), from 17 to 18 days (normal length *LP* = 11; short length *LP* = 4), or after 18 days (normal length *LP* = 5; short length *LP* = 4).

Table 2 presents the predicted population marginal means (LSM) of activity in the normal and short length LP. For univariable and multivariable models, the peak of activity and duration of estrus tended (*P* > 0.05, but < 0.10) to be higher and longer, respectively, with normal length LP than in the short length LP. The one confounder that remained in the multivariable models was BCS, and no variables were statistically significant in the final multivariable models.

**Table 2:** Effect of the duration of the preceding luteal phase (LP) on estrus expression.

	**Descriptive statistics**^**1**^		**Multivariable model**	
	**Peak activity index^3^**	**Duration^4^ (h)**	**Peak activity index^3^**	**Duration^4^ (h)**
**Treatment**				
Normal length LP	82.5 ± 2.7^a^	12.8 ± 0.8	77.3 ± 4.8	11.7 ± 1.1
Short length LP	70.8 ± 5.1^b^	10.9 ± 1.1	69.1 ± 5.1	10.6 ± 1.1
**Parity**				
First	79.9 ± 5.5	13.6 ± 1.1		
Second	80.7 ± 6.8	10.6 ± 1.4		
Third and greater	76.2 ± 4.8	11.7 ± 1.0		
**Milk production (305 days**^2^**)**				
< 11,063 kg	79.5 ± 5.6	12.7 ± 1.2		
11,063–12,520 kg	77.8 ± 6.2	12.7 ± 1.3		
12,520–13,593 kg	76.3 ± 7.2	10.0 ± 1.6		
> 13,593 kg	79.8 ± 7.5	12.3 ± 1.6		
**Body condition score**				
≤ 2.5	65.5 ± 6.8^A^	9.7 ± 1.5^a^	66.9 ± 6.8	9.9 ± 1.5
2.75–3.25	81.5 ± 3.3^B^	12.7 ± 0.7^b^	79.6 ± 3.6	12.4 ± 0.8
**Lameness status**				
Not lame (1 or 2)	79.5 ± 3.6	12.0 ± 0.8		
Lame (3–5)	74.7 ± 6.7	12.6 ± 1.5		

1 *The descriptive statistics present the average (± SD) peak activity index and duration of estrus as detected by a collar-mounted automated activity monitoring system by treatment, parity, milk production, body condition score, and lameness status of 32 cows enrolled in a controlled trial evaluating the difference between normal and short length LP preceding estrus. The multivariable models (one for peak activity index, and one for duration) present the least square means ± SE obtained from the linear regression models*.

2 *Predicted milk production for 305 days at first DHIA test*.

3 *Peak activity index was defined as the highest index value of an estrous event (i.e., period during which the index generated by the AAM was > 35)*.

4 *Duration was defined as the sum of 2 h periods the index generated by the AAM was above the estrus threshold in the system software*.

A, B *Different letters indicate differences between levels of a variable within columns (P < 0.05)*.

a, b *Different letters indicate a tendency for differences between levels of a variable within columns (P > 0.05, but < 0.10)*.

**Figure 5:**
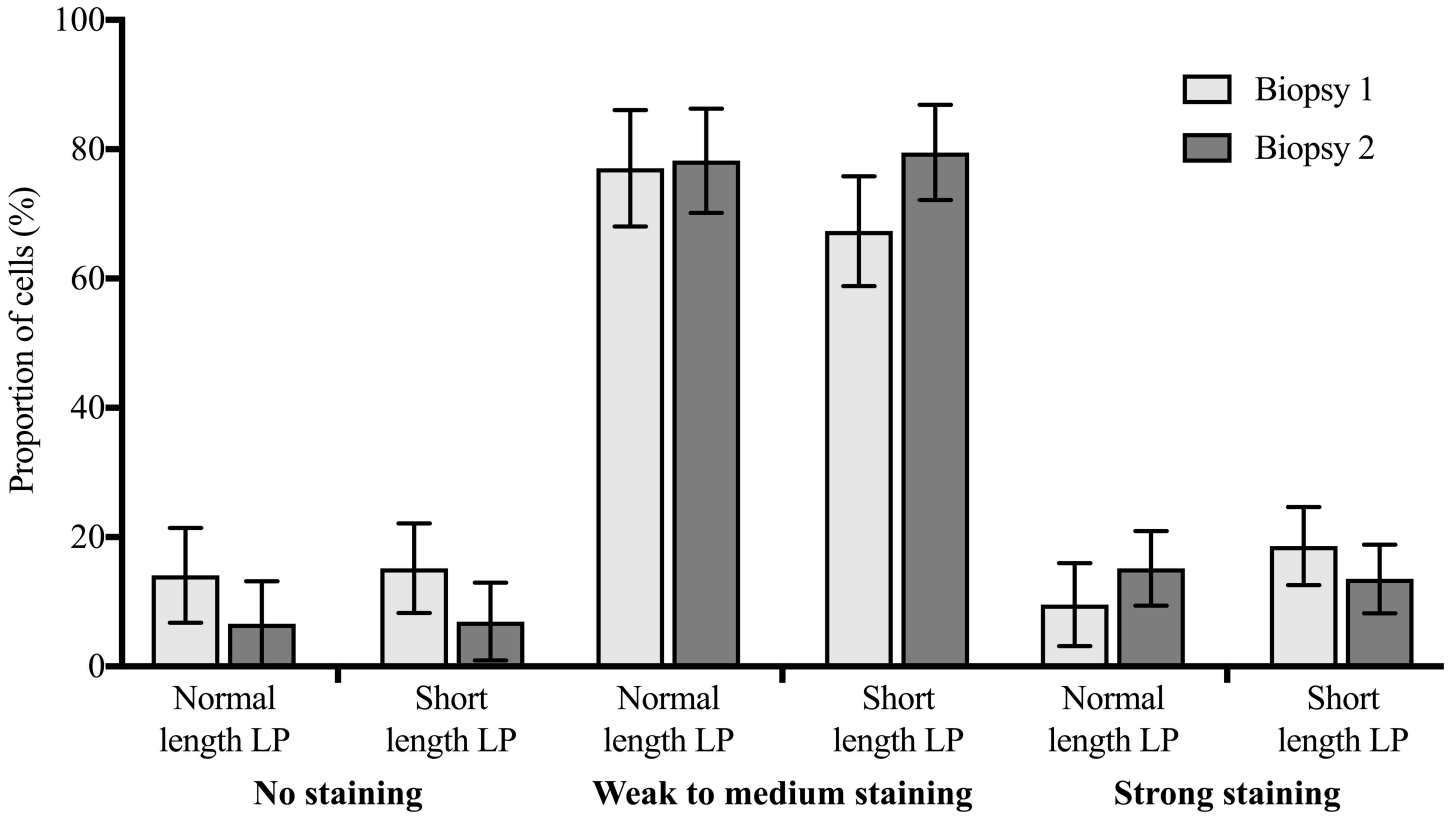
Distribution of immunohistochemical staining for estrogen receptor alpha (proportion ± 95% confidence intervals) in cells from endometrial biopsies 1 (0 days) and 2 (14 days) in 3 staining categories (no staining, weak to medium staining, strong staining), for cows with a short length luteal phase (LP; *n* = 9) or with a normal length LP (*n* = 9) preceding estrus. There was no statistically significant difference between biopsy 1 and 2, or between treatments.

The expression of ERα did not differ by treatment (*P* > 0.10), and there was no difference between biopsy 1 and 2 (*P* > 0.10). Figure 5 presents the predicted population marginal means (LSM) of the proportion of cells in each staining category at biopsy 1 (0 days), and biopsy 2 (14 days), by treatment.

## Discussion

There was no statistically significant difference in peak activity index or duration of estrus between the normal and short length LP. The proportion of cows that did not synchronize during the protocol was higher than expected in the short length LP (*n* = 11; 37%), and also higher than in the normal length LP (*n* = 5; 17%). This could have resulted in a selection bias, as the cows included in the analyses might not be representative of the whole group. For example, cows that did not synchronize could have a common attribute that biases the results by not being represented in the final sample (33). Because of the small numbers, we could not identify a statistical difference between cows that did and did not synchronize, but numerically, cows that did not synchronize were of greater parity (third and greater), normal BCS (2.75–3.25), and had high milk yields (≥12,520 kg). Adding these covariates to the models, despite the matching used for treatment allocation, was used to control for this (35). The higher number of excluded cows with short length LP resulted mainly from cows that did not regress their CL after the PGF_2α_ at 9 days (*n* = 7; 23% of the short length LP). It was shown that a second PGF_2α_ 24 h apart increased complete CL regression, and could have decreased the exclusions in this case (36, 37). Due to these losses to follow up, the final models did not include as many cows as required according to the sample size calculation, even after accounting for the 30% anticipated loss to follow up. Numerically, the peak activity index and duration of estrus were greater for cows with normal length LP than with short length LP. It is unclear if the difference is not statistically significant because the length of the LP did not have a meaningful effect on estrus intensity and duration as detected by the AAM system, or because the number of cows ended up being insufficient. *Post-hoc* power calculation is not recommended (33), but intensity and duration had a *post-hoc* power of 96 and 76%, respectively. That said, the magnitude of the numerical differences (for intensity and duration) between the two treatments is likely too small to have a practical impact on the use of AAM systems. Even though the LP preceding estrus is essential in the estrous cycle for luteinizing hormone release and estradiol sensitization (23, 38), the present results do not support the hypothesis that the length of the LP affects the intensity and duration of estrus as detected by AAM systems.

Only a few studies on a limited number of ovariectomized ruminants support the necessity of exposure to some P4 prior to estrus for estrus expression (24, 39). Fabre-Nys and Martin (24) found a larger proportion of ewes showing estrous behavior, as well as more frequent demonstration of estrous behaviors in ewes that received P4 prior to estradiol, compared to those that did not. Cows that received P4 prior to estradiol were also more likely to show estrous behavior than those that did not, but there was no difference in estrous behavior between cows exposed to P4 for 5 days and those exposed for more than 5 days (39). In both studies, the duration of estrus was not addressed. The present study did not use cows with no previous exposure to P4, but normal or short exposure to P4 resulted in similar intensity and duration of estrus. The study design and sample size did not allow us to compare the difference in the proportion of cows identified in estrus by the AAM. The different length of the LP was not associated with the endometrial expression of ERα either. Circulating estradiol and P4 were previously shown to be associated with the expression of ERα (25, 26), but despite differences in circulating P4 in the week prior to estrus, we found no difference in uterine ERα between the normal and short length LP. As the present study was conducted in a commercial farm, there was no cows with no previous exposure to P4. Cows had a normal or short length LP, that was preceded by P4 exposure (CIDR; Figure 1). This could also have had mitigated the impact of the length of the LP on the intensity and duration of the estrus, as well as the expression of uterine ERα.

Circulating P4 concentrations during follicular growth have previously been shown to affect the diameter of the dominant follicle, and the circulating concentration of estradiol at estrus (40), which could affect estrus intensity and duration. In order to prevent this confounding effect, ECP was used to obtain a comparable peak of circulating estradiol between treatment (41). In the present study, BCS ≤ 2.5 was associated with less intense and shorter duration of estrous activity, as detected by an AAM system. Body condition was also reported to be associated with follicular growth and estradiol concentration at estrus (16). The use of ECP at 15 days of the protocol in the present study should have removed the effect BCS has on estradiol concentration. There was however, a significant association between BCS and estrous activity, which suggests that BCS is associated to estrus expression not only by modifying circulating P4 and estradiol concentrations, but also by mechanisms affecting both BCS and estrus expression such as negative energy balance. The association between BCS and estrus expression has been found before (8, 12, 16), but the exact mechanism involved remains unclear.

In conclusion, we did not detect a difference in intensity or duration of estrus as measured by AAM between a normal and a short length LP in dairy cows, but BCS was associated with intensity and duration of estrus.

## Author contributions

The study design was developed by the collaboration of all the authors. The data collection was done by JD-R, BS, and RA. JD-R performed the statistical analyses and wrote the first draft of the manuscript. All authors contributed to manuscript revision, read and approved the submitted version.

### Conflict of interest statement

The authors declare that the research was conducted in the absence of any commercial or financial relationships that could be construed as a potential conflict of interest.

## Abbreviations

AAM: automated activity monitoring
BCS: body condition score
CL: corpus luteum
DIM: days in milk
DHIA: dairy herd improvement association
ERα: estrogen receptor alpha
GnRH: gonadotropin-releasing hormone
IHC: immunohistochemistry
LP: luteal phase
P4: progesterone
PGF_2α_: prostaglandin F_2α_.
